# Individualized endoscopic management strategy for impacting jujube pits in the upper gastrointestinal tract: a 3-year single-center experience in northern China

**DOI:** 10.1186/s12893-020-01008-y

**Published:** 2021-01-06

**Authors:** Ji-Tao Song, Xiao-Hua Chang, Shan-Shan Liu, Jing Chen, Ming-Na Liu, Ji-Feng Wen, Ying Hu, Jun Xu

**Affiliations:** 1 Department of Gastroenterology and Hepatology, The Second Affiliated Hospital of Harbin Medical University, 246 Xue-Fu Road, Nan-Gang District, Harbin, 150086 China; 2 Department of Gastroenterology and Hepatology, The First Hospital of Harbin, Harbin, China; 3 Faculty of Health Service, Logistics University of Chinese People’s Armed Police Force, Tianjin, China

**Keywords:** Endoscopy, Foreign body, Jujube pit, Upper gastrointestinal tract

## Abstract

**Background:**

Impaction of jujube pits in the upper gastrointestinal (GI) tract is a special clinical condition in the northern Chinese population. Endoscopic removal is the preferred therapy, but there is no consensus on the management strategies. We reported our individualized endoscopic strategies on the jujube pits impacted in the upper GI tract.

**Methods:**

In this retrospective study, we included 191 patients (male: 57; female: 134) who presented to our hospital with ingestion of jujube pits between January 2015 and December 2017. Demographic information, times of hospital visiting, locations of jujube pits, endoscopic procedures, post-extraction endoscopic characteristics were analyzed. Management strategies including sufficient suction, repeated irrigation, jejunal nutrition and gastrointestinal decompression were given based on post-extraction endoscopic characteristics and impacted locations.

**Results:**

Peak incidence was in the second quarter of each year (85/191 cases, 44.5%). Among the 191 cases, 169 (88.5%) showed pits impaction in the esophagus, 20 (10.5%) in the prepyloric region and 2 (1.0%) in the duodenal bulb. A total of 185 patients (96.9%) had pits removed with alligator jaw forceps, and 6 (3.1%) underwent suction removal with transparent caps placed over the end of the endoscope to prevent injury on removal of these pits with two sharp painted edges. Post-extraction endoscopic manifestations included mucosal erosion (26.7%), mucosa laceration (24.6%), ulceration with a white coating (18.9%) and penetrating trauma with pus cavity formation (29.8%). All patients received individualized endoscopic and subsequent management strategies and showed good outcomes.

**Conclusions:**

Individualized endoscopic management for impacted jujube pits in the upper GI tract based on post-extraction endoscopic characteristics and impacted locations was safe, effective, and minimally invasive.

## Background

Many patients may suffer from ingestion and impaction of foreign bodies in the upper gastrointestinal (GI) tract [1]. It is estimated that approximately 10%-20% of cases with foreign body ingestion in the upper GI tract may require endoscopic intervention [2], which is considered to be minimally invasive, involving less complications, as well as decline in the medical expenditures and morbidity rates [3]. To our best knowledge, there are great variations for the endoscopic management strategies based on the ingestion of foreign bodies (e.g. animal bones, coins, dental prostheses and food boluses).

**Fig. 1:**
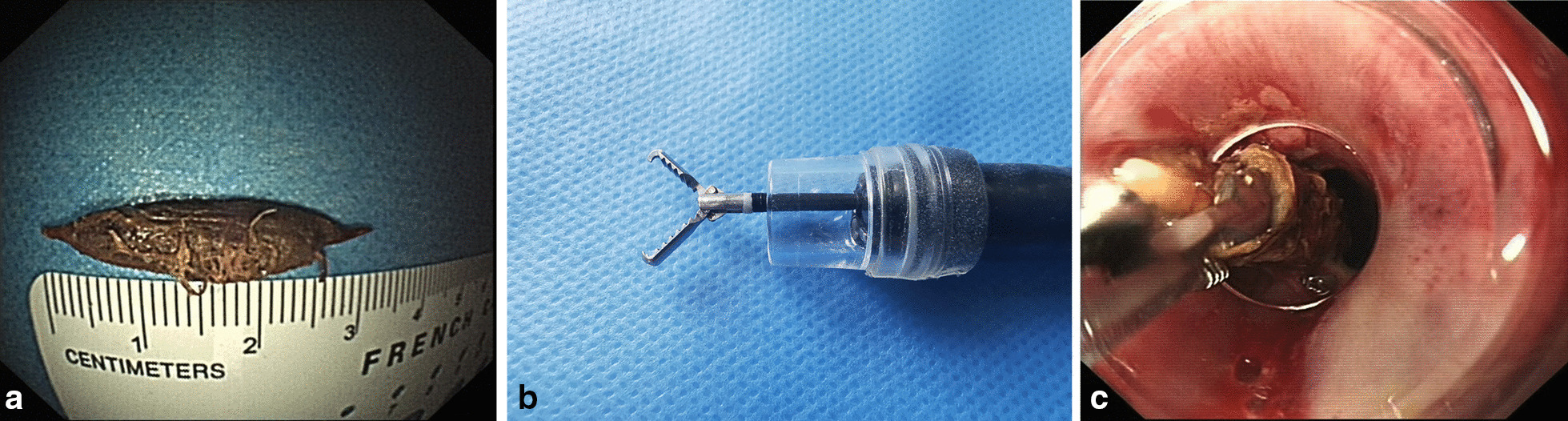
Characteristics of a jujube pit (**a**), transparent cap (**b**) and an endoscopic photo of a pit extracted into the transparent cap (**c**)

Ingestion of jujube pits in the upper GI tract is a common cause for emergency care among the civilians in northern China. Jujube pits from the jujube, a dark red plumlike fruit of Old World buckthorn trees favored by Chinese people usually with two sharp-pointed edges, can penetrate the wall of the GI tract (Fig. 1a). Meanwhile, impaction of jujube pits might lead to several complications, including hemorrhage, fistula and infection. Under rare circumstances, it might be even lethal [4]. On this basis, emergency endoscopic intervention should be given to the patients with ingestion of jujube pits in upper GI tract [5]. However, it is still a challenge to remove the jujube pits from the upper GI as the pits presented two sharp-pointed edges. In addition, there is no consensus on the management strategies upon endoscopic extraction, especially on the management of upper GI penetration trauma with local pus cavity formation. In this study, we reported our experiences on the endoscopic management of jujube pits in the upper GI tract.

## Methods

### Ethics statements

This study was approved by the Ethical Committee of the Second Affiliated Hospital of Harbin Medical University. Written informed consents were obtained from all adult patients and the guardians of paediatric patients.

### Patients

Patients who visited our department with a complaint of dysphagia or irritation, induced by ingestion of jujube pits, between January 2015 and December 2017 were included in this study. Prior to the endoscopic procedures, the following patients were required to undergo computed tomography (CT) to evaluate the potential penetrating injuries and the infection at the serosal side of the involved segment of the gut: those with embedding of jujube pits of ≥ 24 h, or those with suspected complications, or those with challenges in the endoscopic extraction procedures. After removal of the pits under endoscopic guidance, the following information was recorded and analyzed, including demographic characteristics, location of the jujube pit, endoscopic procedures, post-extraction endoscopic characteristics, and post-procedure managements and outcomes.

### Endoscopic procedures

**Fig. 2:**
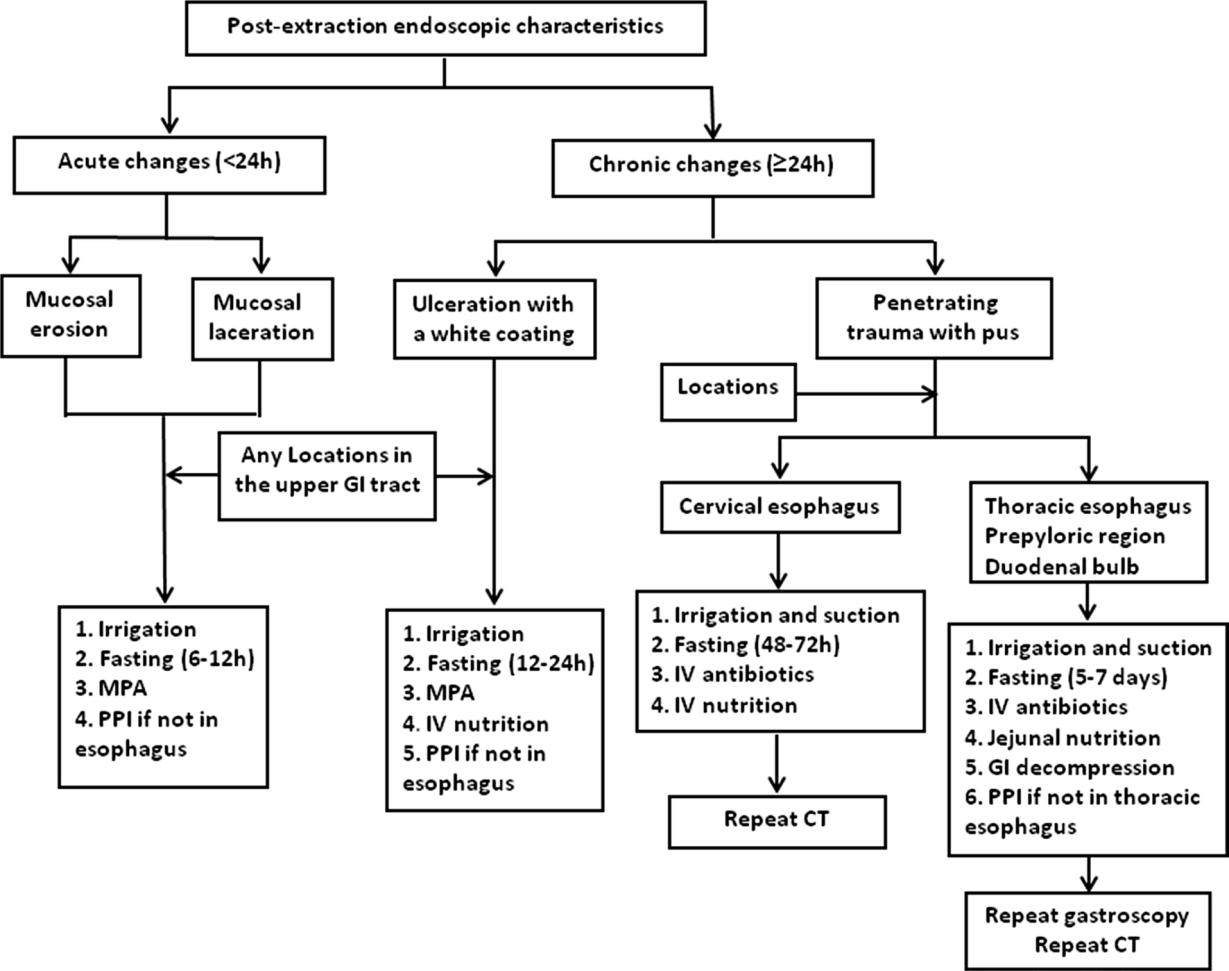
Individualized management strategies during and after endoscopic procedure according to post-extraction endoscopic characteristics and locations. MPA: Mucosal protective agents (aluminium phosphate gel); IV: Intravenous; PPI: Proton pump inhibitor; GI: Gastrointestinal

A standard single-accessory-channel endoscope (GIF-H260, Olympus) with a transparent cap (D-201-11802, Olympus) attached to the tip was utilized for the endoscopic observation. Endoscopic alligator jaw forceps (FG-47L-1, Olympus) were used for the extraction of jujube pits (Fig. 1b). The heart rate, blood pressure, and oxygen saturation for each patient were monitored. After removal of jujube pits, repeated endoscope insertions were required to evaluate the local injuries on the upper GI tract wall. Individualized post-procedure managements were developed based on the injury severity and the impacted position of the pits (Fig. 2).

### Image record and statistical analysis

The post-extraction endoscopic characteristics were recorded by a sophisticated endoscopist in order to avoid observational bias. Upon removal of the pits, attention should be paid to the injury of the digestive tract wall induced by the sharp edges. The injuries induced by both sharp edges were recorded, and the injury extent was evaluated based on the most severe side. The mucosal erosion was defined as superficial injury of mucous membrane, not penetrating the muscularis mucosa layer, with a length of less than or equal to 0.5 cm. Laceration was defined as acute mucous membrane injury reaching or surpassing the muscularis mucosa layer with an injury lesion of more than 0.5 cm in length. Ulceration is defined as chronic mucous membrane injury surpassing the muscularis mucosa layer with white coating. The “penetrating trauma" is transmural erosion with a contained perforation and pus cavity formation. Data analysis was conducted using the SPSS 19.0 software (Chicago, IL, USA). Enumeration data were expressed in frequency.

## Results

### Patients’ characteristics

In total, 191 patients (male: 57; female: 134) were included in this study. The patients were in an age range of 6–92 y, including 103 elder cases (53.9%) with an age of ≥ 60 y, and 88 cases (46.1%) of less than 60. There was a significantly seasonal difference in the incidence of jujube pit impaction in the upper GI tract, with a peak incidence in the second quarter annually (44.5%), followed by the first quarter (22.0%), the third quarter (15.2%) and the last quarter (18.3%), respectively (Table 1).

**Table 1:** Characteristics of patients with jujube pits impacted in the upper GI tract

Parameters	Patients, n	Percentage, %
Sex		
Male	57	29.8
Female	134	70.2
Age		
< 60 years	88	46.1
≥ 60 years	103	53.9
Seasonal characteristics		
First quarter	42	22.0
Second quarter	85	44.5
Third quarter	29	15.2
Fourth quarter	35	18.3
Location		
Cervical esophagus (≤ 18 cm from incisor)	121	63.4
Upper thoracic esophagus (> 18 cm, ≤ 24 cm)	36	18.8
Mid thoracic esophagus (> 24 cm, ≤ 32 cm)	12	6.3
Distal esophagus (> 32 cm to Z-line)	0	0
Prepyloric region	20	10.5
Duodenal bulb	2	1.0
Transparent cap-assisted endoscopic management		
Extract with alligator jaws forceps	185	96.9
Extract by suction	6	3.1
Post-extraction endoscopic characteristics		
Mucosal erosion	51	26.7
Mucosal laceration	47	24.6
Ulceration with a white coating	36	18.9
Penetrating trauma with pus cavity formation	57	29.8

Among the 191 patients, 169 (88.5%) showed pits impaction in the esophagus, including 121 (63.4%) impacted in the cervical esophagus (≤ 18 cm from incisor), 36 (18.8%) in the upper thoracic esophagus (> 18 cm, ≤ 24 cm), 12 (6.3%) in the midthoracic esophagus (> 24 cm, ≤ 32 cm) and 0 (0%) in distal esophagus (> 32 cm to Z-line). Besides, 20 (10.5%) had pits impacted in the prepyloric region and 2 patients (1.0%) with pits impacted in the duodenal bulb.

In this study, endoscopic extraction of jujube pits from the upper GI tract was carried out using two methods. Specifically, 185 (96.9%) received endoscopic transparent cap-assisted extraction of all pits using alligator jaw forceps **(Fig. ****1****C)**, while the other 6 cases (3.1%) received pit extraction by suction using a transparent cap attached to the gastroscope.

### Endoscopic findings of the jujube pits impaction

**Fig. 3:**
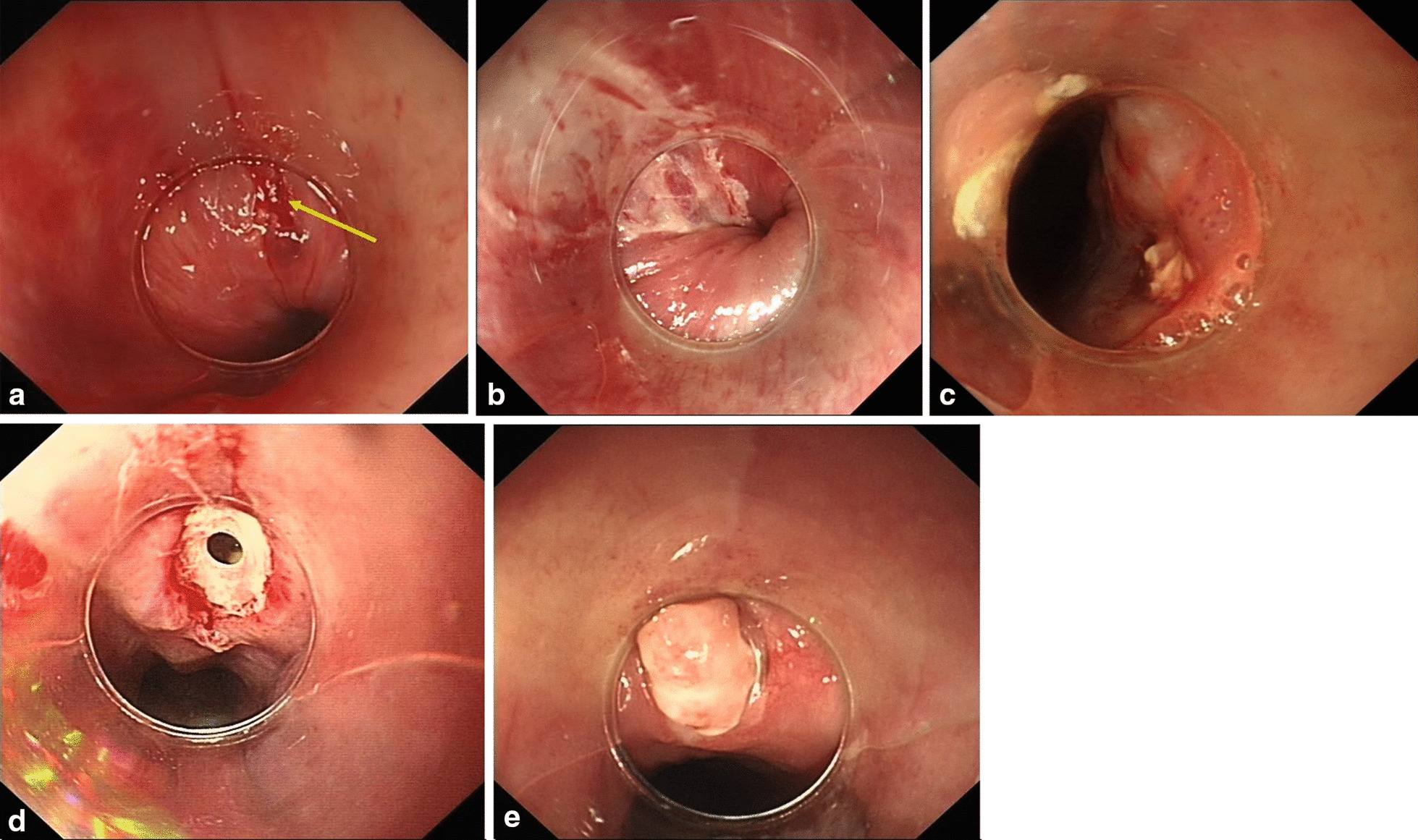
Endoscopic findings of the patients. **a** Endoscopic view of mucosal erosion after removal of jujube pit. **b** Endoscopic view of mucosal laceration. **c** Endoscopic view of ulceration with a white coating. **d** Endoscopic view of penetrating trauma with pus cavity formation. **e** Repeat gastroscope showed the healing of penetrating injury after conservative treatment

Under the endoscopic guidance, post-extraction endoscopic features were as follows: (i) acute changes, defined as local injuries, such as mucosal erosion (Fig. 3a) and mucosal laceration (Fig. 3b) caused by the jujube pits about less than 24 h previously; and (ii) chronic changes, defined as local pathologic changes, such as ulcers with a white coating (Fig. 3c) or penetrating trauma with pus cavity formation (Fig. 3d) presenting for longer than 24 h. Individualized endoscopic and subsequent management strategies were based on different endoscopic manifestations and the different impacted location (Fig. 2). Repeat gastroscopy (Fig. 3e) or CT was performed to confirm healing of the upper GI penetrating injury if necessary.

All the cases underwent endoscopic intervention successfully. There was no mortality associated with the endoscopic procedures in our study. There were variations in the post-extraction endoscopic manifestations including mucosal erosion (26.7%), mucosa laceration (24.6%), ulcer with a white coating (18.9%) and penetrating trauma with pus cavity formation (29.8%). All patients with complications were managed using conservative therapies including endoscopic sufficient suction, repeated irrigation, jejunal nutrition and gastrointestinal decompression. Gastrointestinal decompression tube and naso-jejunal tube have been commonly utilized in treating penetrating trauma with pus cavity formation in the non-cervical esophagus for GI local decompression and jejunal nutrition. In this study, such treatment strategy was utilized to those with involvement of upper thoracic esophagus (n = 12), mid thoracic esophagus (n = 6), and prepyloric region (n = 7). After such treatment, no additional surgical intervention was given. All patients were followed up for two months after discharge.

## Discussion

The primary causes of impaction of jujube pits might be accidental ingestion [6]. Besides, jujube pits impaction in the upper GI tract appeared to be more common in aged patients. To our best knowledge, this is the largest retrospective study on the endoscopic management of jujube pit impaction in the upper GI tract. Meanwhile, individualized management strategies were established based on the anatomic location of the impaction and any associated injury. In the present study, all the 191 cases underwent endoscopic intervention successfully. Additionally, there was a difference in the incidence of jujube pit impaction between different sex. Similarly, Guo et al. [7] reported similar trends in a previous study, in which the prevalence of jujube pit impaction in the female was higher than that of male counterparts. We speculated that it might be related to the fact that women usually consume more jujubes.

Similar with the previous description [8], our data showed that esophagus was the most common impaction location of jujube pits (88.5%). This may be related to the nature of jujube pits as well as the anatomical characteristics of the esophagus. Esophageal impaction might be associated with a particularly high risk of lethal complications [9]. Therefore, emergency endoscopic intervention is usually required in the presence of sharp-pointed objects (e.g. jujube pits) impacting in the esophagus. According to our clinical experiences, even though the pits passed through the esophagus, there was a possibility of impacting in the pylorus and the duodenum. Our results confirmed that even if jujube pits passed through the esophagus spontaneously, they could still impact in the prepyloric region (10.5%) or duodenal bulb (1.0%). Therefore, non-emergency endoscopic intervention is necessary after accidental ingestion of jujube pits even if the jujube pit is not impacted in esophagus after accidental ingestion of jujube pits even if the jujube pit is not impacted in esophagus because pulling the jujube pit into the lumen of this cap prevents any injury on removal of the pit.

The prevalence of ingested foreign bodies is partly depending on the eating habits and socioeconomic context [10]. The prevalence of jujube pit impacted in the upper GI tract among the population in northern China had an obvious seasonal variation, with the peak prevalence in the second quarter annually. This was highly associated with the eating habits and the local culture features. For example, a traditional Chinese festival called the Dragon Boat Festival is held in the second quarter. Many civilians would eat the jujube-containing food, which may induce a high risk of jujube pits impacting in the digestive tract. Thus, special attention and education should be given in order to avoid the accidental ingestion of the pits.

In our study, most of the cases (96.9%) were managed with a transparent cap attached to the tip of a gastroscope and extracted with alligator jaw forceps, while the others (3.1%) were treated by suction through the transparent cap. According to our experiences, cases with small-sized jujube pits impacted the entrance of the esophagus could be effectively managed by suction within a short time. Transparent cap during the endoscopic procedure was an ideal method as previously reported [11]. In our study, the transparent cap also played a vital role in the management of the jujube pits in the upper GI tract. With a transparent cap attached to the gastroscope, the narrow physiological area allowed a clear view and identification of the side of the jujube pits that pierced the GI tract wall [12]. During the procedures, the impacting position of the pits in the digestive tract wall could be observed through the cap at the anterior part of the endoscope. Then a forcep was utilized to grasp the jujube pits, followed by changing the direction of the impaction pits for the extraction of jujube pits. Moreover, transparent cap-assisted endoscopy contributed to the precise assessment of post-extraction endoscopic characteristics and identification of penetrating trauma. This was beneficial to establish the individualized management strategy.

There was significant variation in the post-extraction endoscopic manifestations. In clinical settings, we categorized all endoscopic findings into acute and chronic changes. We proposed individualized management strategies during and after the endoscopic procedures according to the post-extraction endoscopic characteristics and impacted locations. Many patients would develop acute changes within 24 h including mucosal erosion and mucosa laceration as the sharp edges might lead to damages in the mucosa or pierce the muscularis mucosa directly. The sustained impaction of pits in the digestive tract wall would lead to generation of ulcers with a white coating. For the acute changes and ulcers induced by pit impaction, we proposed complete endoscopic irrigation and administration of aluminum phosphate gel for protecting the esophageal tract. For the lesions in the non-esophageal locations, administration of proton pump inhibitors (PPIs) was recommended. A fasting state for 6–12 h was proposed to the cases with acute lesions in the esophagus. For those with ulcer formation, a fasting state for 12–24 h was recommended and intravenous support should be given if necessary.

Under some circumstances, impacted jujube pits might slowly pierce muscularis propria layer of upper GI wall associated with pyogenic infection [13], which then led to penetrating trauma combined with pus cavity formation. Penetrating trauma in the upper GI tract, especially in the esophagus, was a catastrophic condition with a high mortality rate [14]. Upon perforation, appropriate treatment must be given immediately to reduce the mortality and complications. Previously, aggressive surgical approaches, instead of conservative therapies, were recommended for treating such condition as increased mortality was associated with delayed surgery due to conservative therapy [15–17]. Ever since the introduction of self-expandable metallic stents in 2008 [18], stent-based treatment for esophageal perforation has been confirmed to reduce the mortality and increase the healing rates in large-scaled studies [19, 20]. In addition, previous study reported small, contained perforations could be treated conservatively without ongoing leaks on occasion [21].

In our study, all the 57 patients with penetrating trauma showed satisfactory responses to the management strategies with no necessity for delayed surgery or stent-based treatment. Our management strategies varied by impacted location. For pits located in the cervical esophagus, sufficient suction and repeated irrigation at the injury site were essential. In addition to strict dietary restriction and intravenous nutrition, broad-spectrum antibiotics was also administered. Compared with cervical esophagus perforations, thoracic esophageal, prepyloric region and duodenal bulb perforations were relatively difficult to manage because of the different anatomical structures involved and extensive systemic complications. Under those conditions, GI decompression and jejunal nutrition were crucial for local healing, especially in the thoracic esophagus. Although there was a possibility of severe mediastinal infection upon perforation of the thoracic esophagus, our experience indicated that even though there was contained perforation, the local infection was restricted by the healing process of peri-esophageal tissues. In our study, the infection was usually localized rather than diffused and could be effectively managed through conservative treatment.

There are some limitations in our study. Firstly, we could not define the duration of pit impaction or the accurate time to impaction after ingestion. In a recent study [22], impaction of pits in the esophageal tract for more than 24 h had been proposed as an independent risk factor for the penetrating injuries, as it may lead to chronic lesions in the esophageal tract, local ulcer and even formation of fistula in the esophageal tract. Secondly, we did not compare the differences between impaction of pits in the esophageal and non-esophageal tract due to rarity of cases with impaction of pits in the non-esophageal tract. In future, we will focus on the large-sample sized studies to verify the differences.

In summary, the ingestion of jujube pits was more common in female with peak incidence in the second quarter annually in northern China. We proposed individualized management strategies during and after endoscopic procedures according to post-extraction endoscopic characteristics and impacted locations. The strategies were proved to be safe and effective in handling jujube pit impacted in the upper GI tract.

## Data Availability

The datasets used and/or analysed during the current study are available from the corresponding author on reasonable request.

## Abbreviations

GI: Gastrointestinal
CT: Computed tomography
